# Paternal race/ethnicity and very low birth weight

**DOI:** 10.1186/s12884-014-0385-z

**Published:** 2014-11-19

**Authors:** Kimberly G Fulda, Anita K Kurian, Elizabeth Balyakina, Micky M Moerbe

**Affiliations:** Department of Family Medicine, North Texas Primary Care Practice Based Research Network (NorTex), Texas Prevention Institute, University of North Texas Health Science Center, 855 Montgomery, Fort Worth, TX 76107 USA; Tarrant County Public Health, Fort Worth, Texas USA; Texas College of Osteopathic Medicine, University of North Texas Health Science Center, Fort Worth, Texas USA

**Keywords:** Very low birth weight, Maternal and child health, Birth outcomes

## Abstract

**Background:**

The purpose was to examine the association between paternal race/ethnicity and very low birth weight stratified by maternal race/ethnicity.

**Methods:**

Birth data for Tarrant County, Texas 2006–2010 were analyzed. Very low birth weight was dichotomized as yes (<1,500 g) and no (≥1,500 g). Paternal race/ethnicity was categorized as Caucasian, African American, Hispanic, other, and missing. Missing observations (14.7%) were included and served as a proxy for fathers absent during pregnancy. Potential confounders included maternal age, education, and marital status, plurality, previous preterm birth, sexually transmitted disease during pregnancy, smoking during pregnancy, and Kotelchuck Index of prenatal care. Logistic regressions were stratified by maternal race/ethnicity. Odds ratios and 95% confidence intervals were calculated.

**Results:**

Of 145,054 births, 60,156 (41.5%) were Caucasian, 22,306 (15.4%) African American, 54,553 (37.6%) Hispanic, and 8,039 (5.5%) other mothers. There were 2,154 (1.5%) very low birth weights total, with 3.1% for African American mothers and 1.2% for all other race/ethnicities. Among Caucasian mothers, African American paternal race was associated with increased odds of very low birth weight (OR = 1.52; 95% CI:1.08-2.14). Among Hispanic mothers, African American paternal race (OR = 1.66; 95% CI:1.01-2.74) and missing paternal race/ethnicity (OR = 1.65; 95% CI:1.15-2.36) were associated with increased odds of very low birth weight.

**Conclusions:**

Paternal race/ethnicity is an important predictor of very low birth weight among Caucasian and Hispanic mothers. Future research should consider paternal race/ethnicity and further explore the association between paternal characteristics and very low birth weight.

## Background

Marked decreases in infant mortality rates were observed in the United States during the 20th century [1], an indicator often used as an approximation for the general health of a population, and maternal and child health [2]. However, declines in infant mortality have more recently slowed [1]. Infant mortality rates and neonatal mortality rates decreased from 1983 to 2005 with concurrent increases in very low birth weight infant deaths rising from 42.9% to 54.8%. Low birth weight (<2,500 grams) is one of the leading causes of death in the United States [3] and contributed to a lack of decrease in infant and neonatal mortality rates from the years 2000 to 2005 [1]. Low birth weight, including very low birth weight (<1,500 grams), shows disparate distribution across racial/ethnic groups. Almost three percent of births among non-Hispanic African American women were of very low birth weight in 2010, compared to 1.16% among non-Hispanic Caucasian women and 1.20% among Hispanic women [4]. Although the pathway to racial/ethnic disparities in birth outcomes is not completely understood [5-9], recent research has emphasized the need for a more comprehensive understanding of paternal influences on racial/ethnic birth disparities [10,11].

Low birth weight has been associated with paternal age, height, low birth weight, heavy/prolonged lead exposure, and may potentially be related to paternal educational level [12]. Abel and colleagues found that infants born to fathers under twenty years of age were at higher risk of low birth weight compared to fathers between the ages of 20 to 25 years [13]. Similar associations have been found for infant mortality and pre-term birth [13,14]. Paternal lifestyle bears influence on fetal growth through factors such as smoking and the presence of pre-clinical insulin resistance [15]. Paternal involvement during pregnancy and in the infant years has been linked with increased use of prenatal care and maternal abstinence from alcohol and smoking, suggesting additional ways in which paternal variables may affect racial/ethnic disparities [16].

Infant vital statistics are tabulated by maternal race/ethnicity, and many studies of racial/ethnic disparities in birth outcomes follow suit [4,10,17]. Current studies may exclude the potential role of the father in birth outcomes and the complexity of multi-ethnic and multi-racial identity in health disparities research [10]. Outside of the maternal race or ethnicity listed on a birth certificate, an infant may be identified by the race or ethnicity of the father, mother, or both. Furthermore, studies that examine paternal factors in birth outcomes tend to use surrogate markers for paternal influence such as marital status or the presence of a father’s name on a birth certificate [11,18,19].

The United States is expected to move towards a majority-minority population by the year 2043 with expected changes in interracial unions and childbirth [20,21]. Changes in racial/ethnic dynamics have potential implications for birth outcomes [22]. In a retrospective cohort analysis of fetal death, low birth weight, and premature birth among parents with the same or different racial groups, couples with a Caucasian mother and African American father had 1.84 higher odds of stillbirth compared to couples with a Caucasian mother and father. The increased risk of stillbirth was in great part attributable to higher rates of low birth weight and premature births. Increasingly greater risk of stillbirth was found among couples with an African American mother and Caucasian father (OR = 2.01), and couples with an African American mother and father (OR = 2.11) (22). A meta-analysis of stillbirth, preterm birth, and low birth weight of multiracial couples similarly identified significant adverse birth outcomes among biracial couples compared to Caucasian parents, with the greatest risk of negative pregnancy outcomes among African American couples [23]. There appears to be a graded risk for low birth weight among infants born to multiracial couples showing a greater maternal contribution to overall risk.

Few studies examine racial/ethnic disparities in birth weight among infants born to multiracial families of Hispanic origin [24]. Analysis of data from the Fragile Families and Child Wellbeing Study has found that 8% of infants born to mothers of Hispanic origin and African American fathers were low birth weight compared to 6% of infants born to parents who were both of Hispanic origin. Birth outcomes among infants born to African American mothers and Hispanic fathers were closer to outcomes of infants born to African American parents. Birth outcomes among non-Hispanic Caucasian and Hispanic parents did not differ by race/ethnicity.

Although maternal race/ethnicity may contribute more greatly to birth outcomes, paternal race/ethnicity merits closer examination and is not always considered in health disparities research [22-24]. The purpose of this study was to examine the association between paternal race/ethnicity and very low birth weight stratified by maternal race/ethnicity. We hypothesized that paternal race/ethnicity would be associated with VLBW differently for each maternal race/ethnicity.

## Methods

A cross-sectional analysis of birth data for Tarrant County, Texas from 2006–2010 was performed. Live birth data were obtained from the Texas Department of State Health Services Vital Statistics Bureau. A total of 145,054 births were included. Data analysis was approved by the UNT Health Science Center Institutional Review Board.

### Dependent variable

The outcome of interest was very low birth weight (VLBW). VLBW was dichotomized as yes for birth weights <1,500 grams and no for birth weights ≥1,500 grams.

### Independent variables

Maternal and paternal characteristics were examined as risk factors for VLBW. These risk factors included maternal race/ethnicity (Caucasian non-Hispanic, African American non-Hispanic, Hispanic, and other non-Hispanic), maternal age (<20 years, 20–39 years, ≥40 years), maternal education (< high school, high school, > high school), maternal marital status at birth (married, not married), plurality (single, multiple), previous preterm birth (yes, no), sexually transmitted disease (STD) during pregnancy (gonorrhea, syphilis, chlamydia, hepatitis B, or hepatitis C) (yes, no), smoking during pregnancy (yes, no), the Kotelchuck Index of prenatal care (adequate plus, adequate, intermediate, and inadequate), and paternal race/ethnicity (non-Hispanic Caucasian, non-Hispanic African American, Hispanic, non-Hispanic other, and missing). Due to the high number of missing data for paternal race (14.7%), missing observations were included as a level of paternal race/ethnicity. This allowed the data for those individuals to be included in the regression models and served as a proxy for fathers absent during the pregnancy. The Kotelchuck Index, also called the Adequacy of Prenatal Care Utilization (APNCU) Index, combines two crucial elements obtained from birth certificate data, when prenatal care began (initiation) and the number of prenatal visits from when prenatal care began until delivery (received services), into a single summary score. It does not measure the quality of prenatal care and is preferable to other indices because it includes a category for women who receive more than the recommended amount of care (adequate plus) [25].

### Statistical analysis

All analyses were performed with SPSS version 19.0 for Windows software. Descriptive statistics are provided for variables by VLBW (yes or no). Differences in variables by VLBW status were calculated using chi-square analysis (Table 1). Table 2 provides descriptive statistics of maternal and paternal factors for VLBW stratified by maternal race/ethnicity.

**Table 1:** **Sample characteristics, live births, Tarrant County, 2006–2010 (n = 145,054)**

	**Total**	**Birth weight <1,500 grams**	**Birth weight ≥1,500 grams**	**p-value**
	**(n = 145,054)**	**(n = 2,154)**	**(n = 142,886)**	
	***n (%)***	***n (%)***	***n (%)***	
**Maternal Race/Ethnicity**				<0.001
Caucasian, non-Hispanic	60,156 (41.5)	708 (1.2)	59,442 (98.8)	
African American, non-Hispanic	22,306 (15.4)	689 (3.1)	21,615 (96.9)	
Hispanic	54,553 (37.6)	659 (1.2)	53,892 (98.8)	
Other, non-Hispanic	8,039 (5.5)	98 (1.2)	7,937 (98.8)	
**Maternal Age**				<0.001
< 20 years	17,117 (11.8)	290 (1.7)	16,827 (98.3)	
20-39 years	124,865 (86.1)	1,792 (1.4)	123,061 (98.6)	
≥ 40 years	3,069 (2.1)	72 (2.3)	2,996 (97.7)	
**Maternal Education**				0.008
< High school	37,899 (26.1)	526 (1.4)	37,370 (98.6)	
High school	38,259 (26.4)	628 (1.6)	37,627 (98.4)	
> High school	68,839 (47.5)	996 (1.4)	67,838 (98.6)	
**Kotelchuck Index**				<0.001
Adequate Plus	32,235 (22.2)	915 (2.8)	31,318 (97.2)	
Adequate	53,286 (36.7)	229 (0.4)	53,056 (99.6)	
Intermediate	12,300 (8.5)	212 (1.7)	12,088 (98.3)	
Inadequate	44,841 (30.9)	695 (1.6)	44,142 (98.4)	
**Plurality**				<0.001
Single	140,408 (96.8)	1,623 (1.2)	138,772 (98.8)	
Multiple	4,643 (3.2)	531 (11.4)	4,112 (88.6)	
**Previous Preterm Birth**				<0.001
No	143,220 (98.7)	2,073 (1.4)	141,134 (98.6)	
Yes	1,834 (1.3)	81 (4.4)	1,752 (95.6)	
**STD During Pregnancy**				0.462
No	140,406 (96.8)	2,079 (1.5)	138,313 (98.5)	
Yes	4,648 (3.2)	75 (1.6)	4,573 (98.4)	
**Smoking**				0.002
No	137,652 (94.9)	2,012 (1.5)	135,627 (98.5)	
Yes	7,402 (5.1)	142 (1.9)	7,259 (98.1)	
**Paternal Race/Ethnicity**				<0.001
Caucasian, non-Hispanic	52,356 (36.1)	590 (1.1)	51,763 (98.9)	
African American, non-Hispanic	17,821 (12.3)	487 (2.7)	17,333 (97.3)	
Hispanic	46,894 (32.3)	531 (1.1)	46,362 (98.9)	
Other, non-Hispanic	6,612 (4.6)	79 (1.2)	6,533 (98.8)	
Missing	21,371 (14.7)	467 (2.2)	20,895 (97.8)	
**Mother Marital Status**				<0.001
Married	87,078 (60.0)	1,131 (1.3)	85,940 (98.7)	
Not married	57,970 (40.0)	1,023 (1.5)	56,942 (98.2)	

n = number of live births.

Numbers may not equal 100% due to missing data.

Data source: Texas Department of State Health Services.

**Table 2:** **Sample characteristics, very low birth weight births by maternal race/ethnicity, Tarrant County, 2006–2010 (n = 2,154)**

	**Caucasian**	**African American**	**Hispanic**	**Other**
	**(n = 708)**	**(n = 689)**	**(n = 659)**	**(n = 98)**
	***n (%)***	***n (%)***	***n (%)***	***n (%)***
**Maternal Age**				
< 20 years	58 (1.3)	112 (3.0)	114 (1.3)	6 (1.8)
20-39 years	628 (1.2)	553 (3.0)	527 (1.2)	84 (1.1)
≥ 40 years	22 (1.6)	24 (5.8)	18 (1.8)	8 (3.0)
**Maternal Education**				
< High school	81 (1.4)	123 (3.0)	307 (1.1)	15 (1.4)
High school	180 (1.4)	228 (3.1)	198 (1.3)	22 (1.2)
> High school	447 (1.1)	337 (3.1)	152 (1.3)	60 (1.2)
**Kotelchuck Index**				
Adequate Plus	345 (2.2)	280 (5.6)	243 (2.4)	47 (2.7)
Adequate	87 (0.3)	60 (0.9)	72 (0.4)	10 (0.3)
Intermediate	76 (1.3)	65 (3.2)	55 (1.5)	16 (1.8)
Inadequate	160 (1.4)	251 (3.1)	264 (1.1)	20 (0.9)
**Plurality**				
Single	467 (0.8)	537 (2.5)	541 (1.0)	78 (1.0)
Multiple	241 (10.0)	152 (17.5)	118 (10.5)	20 (8.4)
**Previous Preterm Birth**				
No	695 (1.2)	651 (3.0)	631 (1.2)	96 (1.2)
Yes	13 (2.3)	38 (9.9)	28 (3.4)	2 (4.2)
**STD During Pregnancy**				
No	690 (1.2)	659 (3.2)	634 (1.2)	96 (1.2)
Yes	18 (1.7)	30 (1.8)	25 (1.5)	2 (1.0)
**Smoking**				
No	638 (1.2)	630 (3.0)	648 (1.2)	96 (1.2)
Yes	70 (1.3)	59 (5.5)	11 (1.3)	2 (1.7)
**Paternal Race/Ethnicity**				
Caucasian, non-Hispanic	500 (1.1)	16 (2.5)	54 (1.2)	20 (1.2)
African American, non-Hispanic	46 (2.1)	409 (3.0)	26 (1.8)	6 (1.9)
Hispanic	61 (1.1)	17 (3.5)	449 (1.1)	4 (0.9)
Other, non-Hispanic	14 (1.4)	3 (4.2)	3 (0.9)	59 (1.1)
Missing	87 (1.5)	244 (3.4)	127 (1.6)	9 (1.8)
**Mother Marital Status**				
Married	508 (1.1)	231 (2.9)	313 (1.1)	79 (1.2)
Not married	200 (1.3)	458 (3.2)	346 (1.3)	19 (1.4)

n = number of live births.

Data source: Texas Department of State Health Services.

Percents represent the percent within each group that are very low birth weight.

Simple and multiple logistic regression analyses were conducted stratified by maternal race/ethnicity. All variables were included in the multivariate models. Odds ratios and 95% confidence intervals were calculated (Table 3). Analyses were considered statistically significant at the alpha 0.05 level.

**Table 3:** **Adjusted associations between very low birth weight and selected risk factors by maternal race/ethnicity, Tarrant County, 2006-2010**

	**Caucasian**		**African American**		**Hispanic**		**Other**	
	**n =59,113**		**n =21,873**		**n =53,773**		**n =7,848**	
	**OR**	**95% CI**	**OR**	**95% CI**	**OR**	**95% CI**	**OR**	**95% CI**
**Maternal Age**								
< 20 years old	1.09	0.80 - 1.50	1.21	0.95 - 1.53	1.16	0.92 - 1.45	1.75	0.68 - 4.53
20-39 years old	REFERENCE		REFERENCE		REFERENCE		REFERENCE	
> = 40 years old	1.03	0.66 - 1.62	**2.08**	**1.32 - 3.26**	1.56	0.96 - 2.54	**2.26**	**1.05 - 4.89**
**Maternal Education**								
< High School	**1.36**	**1.01 - 1.82**	0.79	0.62 - 1.02	0.92	0.74 - 1.15	1.42	0.74 - 2.71
High School	**1.39**	**1.14 - 1.70**	1.00	0.82 - 1.21	1.02	0.81 - 1.29	1.33	0.79 - 2.25
> High School	REFERENCE		REFERENCE		REFERENCE		REFERENCE	
**Plurality**								
Single	REFERENCE		REFERENCE		REFERENCE		REFERENCE	
Multiple	**10.67**	**8.95 - 12.71**	**6.95**	**5.65 - 8.55**	**9.89**	**7.96 - 12.29**	**6.59**	**3.77 - 11.52**
**Previous Preterm Birth**								
Yes	1.67	0.92 - 3.02	**3.19**	**2.20 - 4.63**	**2.84**	**1.90 - 4.24**	3.77	0.88 - 16.15
No	REFERENCE		REFERENCE		REFERENCE		REFERENCE	
**STD During Pregnancy**								
No	REFERENCE		REFERENCE		REFERENCE		REFERENCE	
Yes	1.20	0.72 - 2.01	**0.47**	**0.32 - 0.70**	1.18	0.78 - 1.78	0.87	0.21 - 3.65
**Smoking**								
Yes	1.00	0.76 - 1.31	**2.01**	**1.49 - 2.72**	1.03	0.56 - 1.90	1.70	0.40 - 7.22
No	REFERENCE		REFERENCE		REFERENCE		REFERENCE	
**Kotelchuck Index**								
Inadequate	**3.50**	**2.67 - 4.59**	**3.22**	**2.41 - 4.29**	**2.47**	**1.89 - 3.21**	**2.60**	**1.20 - 5.60**
Intermediate	**3.77**	**2.76 - 5.15**	**3.56**	**2.49 - 5.09**	**3.53**	**2.48 - 5.04**	**5.39**	**2.43 - 11.97**
Adequate	REFERENCE		REFERENCE		REFERENCE		REFERENCE	
Adequate Plus	**4.78**	**3.75 - 6.08**	**5.39**	**4.05 - 7.18**	**4.89**	**3.74 - 6.40**	**6.69**	**3.32 - 13.46**
**Paternal Race/Ethnicity**								
Caucasian, non-Hispanic	REFERENCE		REFERENCE		REFERENCE		REFERENCE	
African American, non-Hispanic	**1.52**	**1.08 - 2.14**	1.21	0.72 - 2.04	**1.66**	**1.01 - 2.74**	1.41	0.51 - 3.89
Hispanic	0.95	0.71 - 1.26	1.51	0.74 - 3.09	1.16	0.85 - 1.59	0.73	0.24 - 2.20
Other, non-Hispanic	1.29	0.73 - 2.26	1.80	0.49 - 6.62	0.81	0.25 - 2.64	0.89	0.52 - 1.50
Missing	1.25	0.94 - 1.66	1.36	0.80 - 2.34	**1.65**	**1.15 - 2.36**	1.14	0.42 - 3.08
**Mother Marital Status**								
Married	REFERENCE		REFERENCE		REFERENCE		REFERENCE	
Not married	0.99	0.79 - 1.24	1.25	1.02 - 1.53	1.16	0.97 - 1.39	1.03	0.52 - 2.05

n = number of births included in the model after accounting for missing data.

Data source: Texas Department of State Health Services.

Adjusted models include all variables presented in Table 3.

Bolded values are significant at p<0.05.

There was less than one percent missing data for birth weight, maternal race/ethnicity, maternal age, maternal education, marital status, plurality, previous preterm birth, STD during pregnancy, and smoking during pregnancy. There was 1.6% missing for the Kotelchuck Index.

## Results

The sample characteristics are displayed in Table 1. There were 60,156 (41.5%) Caucasian mothers, 22,306 (15.4%) African American mothers, 54,553 (37.6%) Hispanic mothers, and 8,039 (5.5%) other non-Hispanic mothers. Of all births, 2,154 (1.5%) were VLBW. The percent of VLBW was 3.1% for African American mothers and 1.2% for all other racial/ethnic groups.

A majority of mothers were in the 20–39 years age group (86.1%), and almost half of mothers had more than a high school education (47.5%). Most mothers had a single birth (96.8%), did not smoke during their pregnancy (94.9%), and did not have an STD during their pregnancy (96.8%). Few mothers (1.3%) had a previous preterm birth, and 60% of mothers were married. Approximately a third of mothers had adequate prenatal care based on the Kotelchuck Index (36.7%), while almost another third had inadequate care (30.9%). Paternal race/ethnicity was 36.1% Caucasian, 12.3% African American, 32.3% Hispanic, 4.6% other, and 14.7% missing.

A statistically significant difference by VLBW status was observed for maternal race/ethnicity (p < 0.001), maternal age (p < 0.001), maternal education (p = 0.008), Kotelchuck Index (p < 0.001), plurality (p < 0.001), previous preterm birth (p < 0.001), smoking during pregnancy (p = 0.002), paternal race/ethnicity (p < 0.001), and maternal marital status (p < 0.001). No difference in VLBW status pregnancy was observed by having an STD during the pregnancy. Table 2 provides VLBW by maternal and paternal characteristics stratified by maternal race/ethnicity. Among all maternal racial/ethnic groups, women 40 years of age and older had a higher percentage of VLBW. Additionally, women with a Kotelchuck Index score of adequate plus, multiple plurality, and a previous preterm birth had a higher percentage of VLBW across all maternal racial/ethnic groups. Among Caucasian, Hispanic, and other mothers, African American paternal race had a higher percentage of VLBW. Among African American mothers, other non-Hispanic paternal race had the highest percentage of VLBW.

### Regression findings

In crude analysis, African American mothers had a 2.68 times greater odds (95% CI: 2.41-2.98) of VLBW as compared to Caucasian mothers. Hispanic and other mothers were not significantly different than Caucasian mothers. Adjusted ORs from multiple logistic regression analyses stratified by maternal racial/ethnic groups are displayed in Table 3. The adjusted analysis revealed no association between maternal age and VLBW for Caucasian and Hispanic mothers. In both African American (OR = 2.08; 95% CI: 1.32-3.26) and other (OR = 2.26; 95% CI: 1.05-4.89) mothers, being ≥40 years of age was associated with a greater odds of VLBW as compared to 20–39 years of age. Maternal education was only associated with an increased odds of VLBW among Caucasian mothers with having less than a high school degree associated with a 36% increased odds (OR = 1.36; 95% CI: 1.01-1.82) and having a high school degree or GED associated with a 39% increased odds (OR = 1.39; 95% CI: 1.14-1.70) as compared to having more than a high school diploma.

Multiple plurality was significantly associated with higher odds of VLBW in all racial/ethnic groups, {Caucasians (OR = 10.67; 95% CI: 8.95-12.71), African Americans (OR = 6.95; 95% CI: 5.65-8.55), Hispanics (OR = 9.89; 95% CI: 7.96-12.29), others (OR = 6.59; 95% CI: 3.77-11.52)}. Previous preterm birth was significantly associated with higher odds of VLBW in African American (OR = 3.19; 95% CI: 2.20-4.63) and Hispanic (OR = 2.84; 95% CI: 1.90-4.24) mothers, but not among Caucasian or other mothers. Having an STD during the pregnancy was significantly associated with a lower odds of VLBW among African American mothers (OR = 0.47; 95% CI: 0.32-0.70) but not among the other racial/ethnic groups. Smoking was significantly associated with higher odds of VLBW among African American mothers (OR = 2.01; 95% CI: 1.49-2.72), but not among the other racial/ethnic groups. Among all racial/ethnic groups, inadequate, intermediate and adequate plus Kotelchuck Index categories were significantly associated with a higher odds of VLBW compared to adequate Kotelchuck Index (Table 3). Notably, the adequate plus group had the highest odds ratio for each racial/ethnic group: Caucasian (OR = 4.78; 95% CI: 3.75-6.08); African American (OR = 5.39; 95% CI: 4.05-7.18); Hispanic (OR = 4.89; 95% CI: 3.74-6.40); other (OR = 6.69; 95% CI: 3.32-13.46). Maternal marital status was associated with VLBW among African American mothers (OR-1.25; 05% CI: 1.02-1.53), such that being single was associated with an increased odds of VLBW.

Paternal race/ethnicity was associated with an increased odds of VLBW among Caucasian and Hispanic mothers. Among Caucasian mothers, having an African American father was associated with a 52% increased odds of VLBW (OR = 1.52; 95% CI: 1.08-2.14). Among Hispanic mothers, having an African American father was associated with a 66% increased odds of VLBW (OR = 1.66; 95% CI: 1.01-2.74) and missing father was associated with a 65% increased odds of VLBW (OR = 1.65; 95% CI: 1.15-2.36). Paternal race/ethnicity was not associated with VLBW among African American or other mothers.

## Discussion

Paternal race/ethnicity is an important predictor of very low birth weight among non-Hispanic Caucasian and Hispanic mothers. This relationship is significant apart from marital status and maternal factors such as age, plurality, and smoking. Among non-Hispanic Caucasian mothers, African American paternal race was associated with increased odds of VLBW compared to non-Hispanic Caucasian paternal race. Studies have previously identified a graded increase in risk for stillbirth among infants born to families with non-Hispanic Caucasian maternal and African American paternal race, African American maternal and non-Hispanic Caucasian paternal race, and African American maternal and paternal race, respectively [22]. Similar trends have been identified for pre-term birth and low birth weight [23,24,26]. Notably, the current study did not identify a significant difference in birth outcomes among African American mothers with African American paternal race listed on the birth certificate compared to non-Hispanic Caucasian paternal race. Prior studies have not been able to examine this difference due to lack of sample size [24] or have found greater risk among African-American couples [23,26]. Further research is needed to clarify this difference.

The increased odds of VLBW related to paternal race/ethnicity exist independently of marital status. Sullivan and colleagues found that although infants born to unmarried mothers were at higher risk for low birth weight and pre-term birth than married mothers, this association failed to explain the African American-Caucasian disparity in birth outcomes; however, paternal race/ethnicity was not examined in this study [27]. Paternal race/ethnicity may be a proxy for unmeasured or unidentified mechanisms [22] by which paternity influences birth outcomes apart from marital status. The present study found that marital status was significantly associated with increased odds of VLBW only among African-American mothers. Unmarried African-American women had greater odds of delivering a VLBW infant as compared to married African-American women. Marital status was not associated with VLBW among other maternal race/ethnicities. Significant associations were still found for paternal race/ethnicity among non-Hispanic Caucasian and Hispanic women although marital status was not significantly associated with VLBW.

The present study confirms that the lack of a paternal record is associated with greater odds of VLBW among Hispanic women but not among non-Hispanic Caucasian, African American, or other women. Previous research indicates that unmarried African American, non-Hispanic Caucasian, and Hispanic mothers with no paternal record on the birth certificate are at greater risk of delivering a low birth weight infant when compared to married mothers [19]. Other studies also suggest that the lack of a father on the birth certificate is associated with increased risk for infant mortality, low birth weight, very low birth weight, and pre-term birth [16,18]. The present study considers paternal race/ethnicity in the analysis and may explain differences in study outcomes. Results may also vary based on differences in other factors that were not available in this analysis such as maternal health characteristics (diabetes, cardiac disease, anemia, etc.). The protective role of social support in Hispanic birth outcomes may be compromised or reduced among Hispanic mothers with no paternal record on the birth certificate [28,29].

Factors such as stress, increased susceptibility to stress, the presence of racism and its potential to increase or exacerbate stress, and racial/ethnic differences in biological responses to stressors have been considered possible explanations to racial/ethnic disparities in birth outcomes, each bearing some merit, but lacking in capacity to completely clarify the pathway to differences in health [9]. Longitudinal models suggest that a cumulative understanding of protective and risk factors that result from early life exposures and that wear on a woman’s allostatic system over time may more accurately capture the extent of racial/ethnic disparities in birth weight than snapshots of maternal and infant risk factors during pregnancy alone [8]. Results of this study suggest that future models should consider variables dynamic to the father-mother-infant triad in addition to maternal and infant factors over the lifecourse.

The large sample size and the inclusion of paternal/race ethnicity in the analysis are clearly strengths of this study. However, paternal age was not included in the analysis due to missing data, and other factors such as paternal/maternal income and national origin of Hispanic populations were not included in the analysis. This study is specific to Tarrant County Texas, and results may not be applicable to other study populations. Maternal risk factors for specific medical conditions were not available from the birth certificate data. Additionally, the Kotelchuck Index, also called the Adequacy of Prenatal Care Utilization (APNCU) Index, does not measure the quality of prenatal care. While it is preferable to other indices because it includes a category for women who receive more than the recommended amount of care (adequate plus), the adequate plus category may include complex pregnancies that required more visits. For the current analysis, the adequate category was used as the reference group as opposed to the adequate plus.

## Conclusions

Paternal characteristics are associated with negative birth outcomes; however, the extent of the influential effects of paternal characteristics is not fully understood. Existing evidence supports the continued exploration of paternal factors such as race/ethnicity with VLBW, and programs and policies aimed at improving birth outcomes should include fathers and paternal factors in the provision of services. Other paternal characteristics such as age and education should also be examined in relation to birth outcomes. Finally, it is unclear if the association between paternal race/ethnicity and birth outcomes is reflective of social constructs such as cultural practices or familial/social support that may be better captured by measuring other factors. Future research is needed to understand the stressors that may be unique to multi-ethnic unions and among mothers where paternity is absent, even in the presence of protective factors such as Hispanic maternal ethnicity. Additional research should include a prospective approach to capture family and cultural dynamics before, during, and after pregnancy to clarify these complex relationships.

## Abbreviation

VLBW: Very low birth weight
